# Wall Shear Stress Predicts Media Degeneration and Biomechanical Changes in Thoracic Aorta

**DOI:** 10.3389/fphys.2022.934941

**Published:** 2022-07-07

**Authors:** Miika Kiema, Jaakko K. Sarin, S. Petteri Kauhanen, Jari Torniainen, Hanna Matikka, Emma-Sofia Luoto, Pekka Jaakkola, Petri Saari, Timo Liimatainen, Ritva Vanninen, Seppo Ylä-Herttuala, Marja Hedman, Johanna P. Laakkonen

**Affiliations:** ^1^ A.I. Virtanen Institute for Molecular Sciences, University of Eastern Finland, Kuopio, Finland; ^2^ Department of Clinical Radiology, Kuopio University Hospital, Kuopio, Finland; ^3^ Department of Applied Physics, University of Eastern Finland, Kuopio, Finland; ^4^ Department of Medical Physics, Medical Imaging Center, Pirkanmaa Hospital District, Tampere, Finland; ^5^ Department of Heart and Thoracic Surgery, Kuopio University Hospital, Heart Center, Kuopio, Finland; ^6^ Research Unit of Medical Imaging, Physics and Technology, Oulu University Hospital, Oulu, Finland; ^7^ Science Service Center, Kuopio University Hospital, Kuopio, Finland; ^8^ Gene Therapy Unit, Kuopio University Hospital, Kuopio, Finland; ^9^ Institute of Clinical Medicine, University of Eastern Finland, Kuopio, Finland

**Keywords:** thoracic aortic aneurysm (TAA), wall shear stress, media degeneration, smooth muscle cells, wall strength, 4D flow MRI, biomechanics, MYH10

## Abstract

**Objectives:** In thoracic aortic aneurysm (TAA) of the ascending aorta (AA), AA is progressively dilating due to the weakening of the aortic wall. Predicting and preventing aortic dissections and ruptures in TAA continues to be challenging, and more accurate assessment of the AA dilatation, identification of high-risk patients, and timing of repair surgery are required. We investigated whether wall shear stress (WSS) predicts pathological and biomechanical changes in the aortic wall in TAA.

**Methods:** The study included 12 patients with bicuspid (BAV) and 20 patients with the tricuspid aortic valve (TAV). 4D flow magnetic resonance imaging (MRI) was performed a day before aortic replacement surgery. Biomechanical and histological parameters, including assessing of wall strength, media degeneration, elastin, and cell content were analyzed from the resected AA samples.

**Results:** WSSs were greater in the outer curves of the AA compared to the inner curves in all TAA patients. WSSs correlated with media degeneration of the aortic wall (*ρ =* -0.48, *p* < 0.01), elastin content (*ρ* = 0.47, *p* < 0.01), and aortic wall strength (*ρ =* -0.49, *p =* 0.029). Subsequently, the media of the outer curves was thinner, more rigid, and tolerated lower failure strains. Failure values were shown to correlate with smooth muscle cell (SMC) density (*ρ* = -0.45, *p* < 0.02), and indicated the more MYH10^+^ SMCs the lower the strength of the aortic wall structure. More macrophages were detected in patients with severe media degeneration and the areas with lower WSSs.

**Conclusion:** The findings indicate that MRI-derived WSS predicts pathological and biomechanical changes in the aortic wall in patients with TAA and could be used for identification of high-risk patients.

## 1 Introduction

In the thoracic aortic aneurysm (TAA), ascending aorta (AA) progressively dilates due to the weakening of the aortic wall structure. This can lead to severe complications, such as aortic dissection or rupture. The incidence of TAA is approximately 5*–*10/100,000 persons/year with AA dilatation accounting for ∼60% (Kuzmik et al., 2012). Risk factors for AA dilatation are hypertension, bicuspid aortic valve (BAV), and certain genetic factors and syndromes (Isselbacher, 2005). Typically, patients are asymptomatic, and TAA is found incidentally in chest imaging performed due to other reasons. Surgical intervention is recommended if the aortic diameter exceeds 55 mm in cases with normal aortic valve and without inherited aortic disease due to an increased risk of aortic rupture or dissection (Hiratzka et al., 2010; Erbel et al., 2014). Besides surgical intervention, no effective treatment currently exists for TAA.

The weakening of the aortic wall and media degeneration are the hallmarks of TAA (Milewicz et al., 2008). A clear association has been demonstrated with AA dissection and genetic factors that encode smooth muscle cell (SMC) contraction, adhesion, metabolism, or transforming growth factor-beta (TGF-β) signaling (Guo et al., 2017). However, only ∼20% of TAAs are familial (Pinard et al., 2019). Besides genetics, biomechanical forces have been suggested to cause or induce the progression of the AA dilatation (Davies, 1995). Focal increases in wall shear stress (WSS) (Barker et al., 2012) and higher circumferential WSS (Rodríguez-Palomares et al., 2018) have been detected in BAV patients with TAA compared to healthy subjects. We have also reported flow displacement and increased circumferential WSS in patients with tricuspid aortic valve (TAV) with TAA (Kauhanen et al., 2019; Korpela et al., 2022). Higher shear forces have been suggested to explain the higher prevalence of TAA in patients with BAV in comparison to TAV (Barker et al., 2012; Meierhofer et al., 2013; Mahadevia et al., 2014; van Ooij et al., 2017). Based on our earlier work (Kauhanen et al., 2019) and others (Bürk et al., 2012; van Ooij et al., 2015), in healthy subjects total WSSs are typically greater compared to TAA, whereas circumferential WSS is lower.

To predict aortic rupture, knowledge of the aorta’s biomechanical properties and the ability to sustain stress is crucial. Reduced levels of elastic fibers have been associated with increased aortic wall stiffness in TAA (Iliopoulos et al., 2009b), leading to decreased aortic wall strength (Vorp et al., 2003). Cellular remodeling is suggested to be involved in TAA acting as an adaptive response to minimize increased stress of the aortic wall (Tang et al., 2005). Knowledge of the relation of WSS, cellular changes, media degeneration, and biomechanics is, however, limited and has not been studied in the same patients in detail. We hypothesized that changes in flow conditions alter aortic wall structure and cell content in TAA. To study this, preoperative 4D flow magnetic resonance imaging (MRI) was performed a day before aortic replacement surgery. Biomechanical and histological parameters were analyzed from resected AA samples to estimate the effect of WSS on the aortic wall.

## 2 Materials and Methods

### 2.1 Patient Cohorts

Samples were collected from TAA patients (n = 32, Table 1) during routine elective surgery at Kuopio University Hospital (KUH) between 11/2017 and 02/2020. Surgical treatment was based on clinical practices. Following information concerning patients’ medical history was collected: age, gender, height, weight, surgery indication, hematocrit value before the operation, location and the highest diameter of AA dilatation, aortic valve morphology, preoperative diseases, and medication. Patients with mechanical aortic valve or genetic disorders (known to affect the development of AA aneurysms) were excluded. Prior to surgery, patients’ aortas were imaged with 4D flow MRI.

**TABLE 1:** Patient demographics.

	Patients with TAA *n* = 32	TAV *n* = 20	BAV *n* = 12	Healthy *n* = 4	*p*-value TAV vs BAV
Patient data					
Age (years)	62.9 ± 7.8	65.6 ± 6.8	58.3 ± 7.3	59.3 ± 8.4	0.01
Gender (n of male/female)	26/6	15/5	11/1	3/1	0.4
BMI (kg/m^2^)	28.4 ± 3.5	27.6 ± 3.1	29.7 ± 3.7	N/A	0.1
Patients’ medical history					
Hypertension (n)	22 (69%)	13 (65%)	9 (75%)	2 (50%)	0.6
Dyslipidemia (n)	14 (44%)	8 (40%)	6 (50%)	1 (25%)	0.6
Diabetes (n)	1 (3%)	1 (5%)	0 (0%)	2 (50%)	0.5
Coronary artery disease (n)	3 (9%)	2 (10%)	1 (8%)	0 (0%)	0.9
Medication, statins (n)	16 (50%)	11 (55%)	5 (42%)	0 (0%)	0.5
Aortic and valvular data					
The largest diameter of AA (mm)	53.0 ± 4.9	54.5 ± 4.7	50.6 ± 4.1	36.1 ± 3.9	0.03
Location of the largest diameter of AA (tubular/sinus Valsalva)	21/11	12/8	9/3	N/A	0.5
Aortic regurgitation (n)	19 (59%)	11 (55%)	8 (67%)	0 (0%)	0.5
Aortic stenosis (n)	3 (9%)	1 (5%)	2 (17%)	0 (0%)	0.3
Pre-operative MRI performed (n)	22	13	9	0	0.6
Elasticity measurements performed (n)	23	15	8	4	0.6

Mean and SD, values are presented.

**FIGURE 1:**
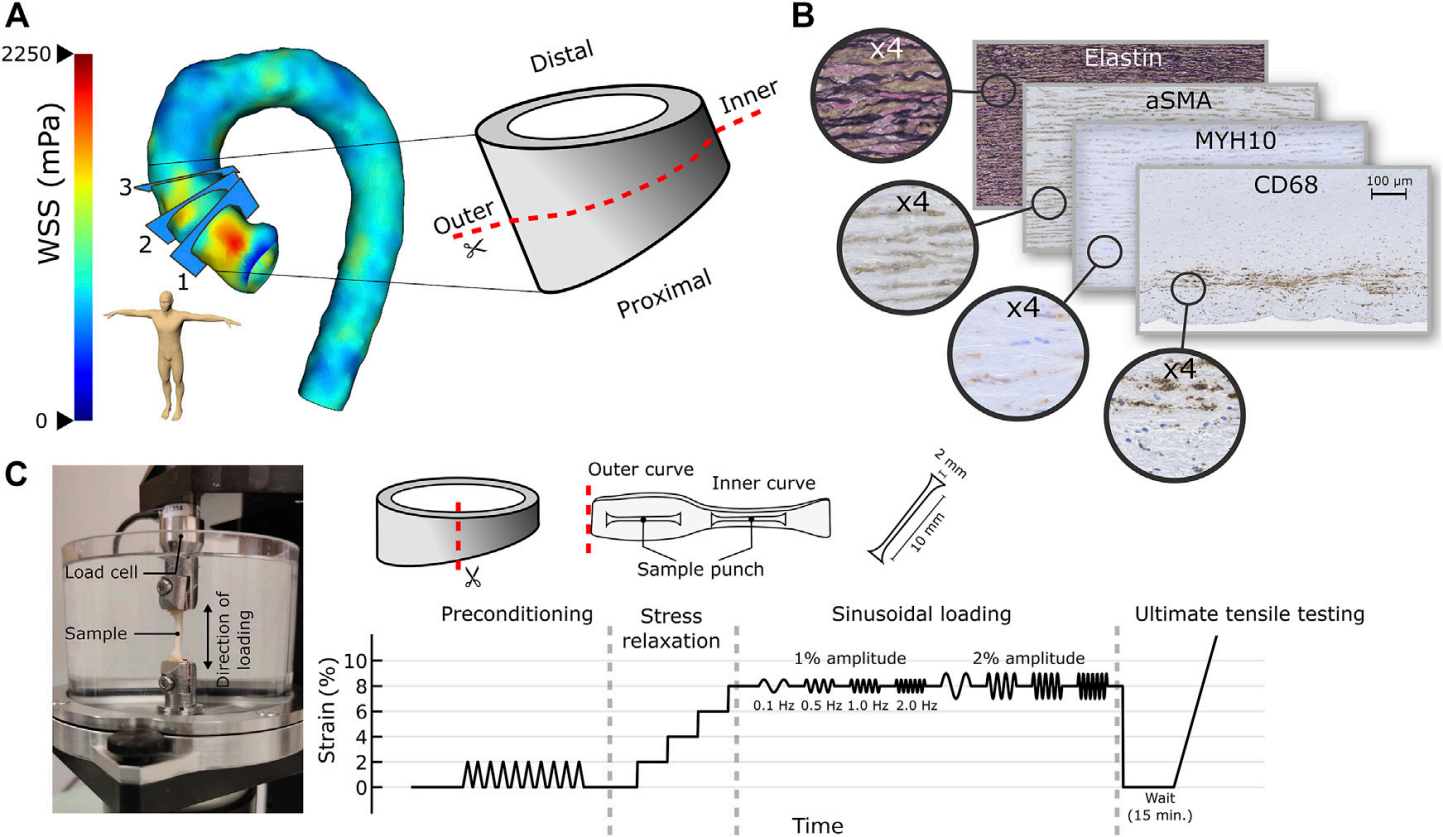
**(A)** A WSS map of dilated AA along with the three analysis planes and the extracted aortic sample subjected to histological (distal part) and biomechanical (proximal part) measurements are presented. **(B)** Media degeneration scoring was performed together with histological stainings to detect elastin, smooth muscle cells (α-SMA, MYH10), and inflammatory cells (CD68, CD3) from the inner and outer curves of the AA. **(C)** Sample processing in the biomechanical measurements along with the measurement system and protocol.

During aortic reconstruction, a part of AA was removed and divided into distal and proximal parts (Figure 1). The outer curves were marked with a thread to track the orientation. The distal part of the aorta was fixed in 4% paraformaldehyde and divided into pieces (∼10–25 depending on size). The inner and outer curve segments were separated for histology. The proximal part of the aorta was immersed in NaCl and refrigerated, followed by post-haste subjection to biomechanical measurements within 24 h. Control samples (n = 4) for biomechanical measurements and histology were collected at KUH from organ donors which had normal aortic dimensions in the chest computed tomography acquired after hospital admission, and no history of major cardiovascular diseases. The resection of AA was performed similarly to the aortic reconstruction operation. The handling of tissue samples was identical for patients and controls.

### 2.2 Magnetic Resonance Imaging of TAA Patients

MR angiography and 4D flow MRI sequences were performed with Siemens MAGNETOM Aera, 1.5 T scanner (Siemens GmbH, Erlangen, Germany) without contrast agent a day before the operation. The total scan time was approximately 45 min.

#### 2.2.1 Sequences

The imaging procedure has been described in detail by Kauhanen et al. (Kauhanen et al., 2019). Briefly, MR angiography (TRUFI with a respiration navigator) was performed to measure the dimensions of the aorta. The AA diameter was measured from the outer-to-outer vascular wall perpendicular to the centerline of the vessel using multiplanar reconstruction. The largest diameter of the AA was registered.

A standard Siemens phase-contrast 4D flow sequence was performed with ECG-gating and in free breathing. The imaging parameters were selected in line with the 4D flow MRI consensus statement (Dyverfeldt et al., 2015).

#### 2.2.2 WSS Analysis

The analysis procedure has been described in detail by Kauhanen et al. (Kauhanen et al., 2019). Briefly, the 4D flow data was analyzed at peak systole with CAAS MR 4D flow 5.0 software (Pie Medical Imaging, Maastricht, Netherlands). Temporal total WSS is defined as a geometric sum of circumferential (parallel to the emitter plane) and axial (perpendicular to the emitter plane) WSSs. The WSS is determined by multiplying wall shear rate with blood viscosity (4 mPas)*.* WSSs were later corrected with patient-specific blood viscosity (Pries et al., 1992). Total and circumferential WSSs were analyzed from planes 1*–*3 (Figure 1A), thereby covering the levels of reference analyses. The software measured WSSs at 4° intervals. The average, minimum, and maximum WSSs were calculated separately for the inner and outer curves from 120° segments.

### 2.3 Histology

Paraffin-embedded aorta samples were sectioned, and elastin staining was performed using Verhoeff-Van Gieson elastic stain kit (Merck, Darmstadt, Germany) according to manufacturer’s protocol. Media degeneration was scored on a scale of 0*–*3 based on Halushka et al. consensus statement (Halushka et al., 2016). Media degeneration parameters, such as elastic fiber fragmentation and/or loss and SMC nuclei loss, were evaluated based on the severity (lesion involves ≤3 lamellar units [mild], 4*–*10 lamellar units [moderate], >10 lamellar units [severe]) and the extent (lesion not present [absent], involvement of the area <10% [focal], multiple areas of 10*–*30% [multifocal], >30% [extensive]) to determine degenerative aortic histopathology (Figure 1B). The amount of elastin per area (i.e., elastin density) and media thickness were analyzed from the inner and outer curve segments (∼120°). Inflammation was based on hematoxylin and eosin staining on a scale of 0*–*3 (0 absent, 1 mild, 2 moderate, 3 severe). Stained tissue sections were imaged with Aperio CS2 slide scanner (Leica Biosystems, Wetzlar, Germany) or Nanozoomer-XR Digital slide scanner (Hamamatsu, Hamamatsu City, Japan) in Biobank of Eastern Finland (Kuopio, Finland). Representative images were taken with Eclipse Ni-E microscope (4×/0.13 and 20×/0.5 Plan Fluor objective; Nikon, Tokyo, Japan). The total amount of elastin per area was analyzed with Aperio ImageScope software (Leica Biosystems). Media thickness was measured with Aperio ImageScope software or NDP. view2 software (Hamamatsu). When correlating WSSs and histological properties, planes 2 and 3 were used. All stainings were performed on both TAA and control samples.

#### 2.3.1 Immunohistochemistry

Tissue sections were blocked with serum-free protein block (X0909, Agilent Technologies, Santa Clara, CA), incubated with primary antibodies and counterstained with Harris’ hematoxylin. In fluorescent stainings, nuclei were stained with DAPI (H-1200, Vector Laboratories, Burlingame, CA). Following primary antibodies were used: monoclonal mouse anti-human smooth muscle actin (α-SMA, M0851, Agilent Technologies; dilution 1:200), monoclonal mouse anti-actin, α-smooth muscle-Cy3 (C6198, Merck; dilution 1:1,000), monoclonal rabbit recombinant anti-non-muscle myosin IIB/MYH10 (ab230823, Abcam, Cambridge, UK; dilution 1:500), monoclonal mouse anti-human CD68 (M0814, Agilent Technologies; dilution 1:100), and monoclonal mouse anti-human CD3 (M7254, Agilent Technologies; dilution 1:100). Biotinylated horse anti-mouse IgG (BA-2000, Vector Laboratories) and biotinylated goat anti-rabbit IgG (BA-1000, Vector Laboratories) were used as secondary antibodies. From alpha-smooth muscle actin (α-SMA) and myosin heavy chain 10 (MYH10) immunostainings, the number of positive cells and protein expression were determined. SMC loss was analyzed and scored on a scale of 0*–*3 (0 no loss, 1 mild, 2 moderate, 3 severe). Change in SMC orientation from linear (parallel to elastic fibers) to random (perpendicular to elastic fibers) was scored on a scale of 0*–*3 (0 linear orientation, 1 mild change, 2 moderate change, 3 random orientation). Areas of MYH10, CD68, and CD3 positive cells were scored on a scale of 0*–*3 (0 absent, 1 low, 2 moderate, 3 high). Imaging was performed by Biobank of Eastern Finland with Nanozoomer-XR Digital slide scanner (Hamamatsu). Representative images were taken with Eclipse Ni-E microscope (10×/0.3 and 20×/0.5 Plan Fluor objective, Nikon). For α-SMA and MYH10 stainings, the quantitation of positive area was performed by NIS-Elements (Nikon) or Aperio ImageScope software (Leica Biosystems). For the analysis of MYH10^+^, CD68^+^, and CD3^+^ cells, the media was divided into three equally sized layers (inner media i. m. middle media m.m. and outer media o. m.). MYH10 area was also analyzed from the intimal layer. In addition, CD68 and CD3 were scored from the intimal layer and adventitia. When correlating WSSs and immunohistochemical properties, planes 2 and 3 were used. All stainings were performed on both TAA and control samples.

### 2.4 Biomechanical Measurements

Two circumferential dumbbell-shaped specimens were cut with a custom punch tool from the inner and outer curves of the AA from TAA and control patients (Figure 1C). Specimen’s physical dimensions were determined with a caliper. A commercial mechanical tester (Mach-1 v500css, Biomomentum Inc. Laval, Canada) was used in the uniaxial testing of the specimen. Double-sided sandpapers (Mirox P80, Mirka Oy, Uusikaarlepyy, Finland) were glued (Loctite Precision, Henkel AG, Düsseldorf, Germany) on both ends and sides of the samples to avoid slippage from the testing clamps. Two load cells (17N, MA239, and 250N, MA297, Biomomentum Inc.) were used for the testing of the specimen. The measurement protocol consisted of a preload and preconditioning, followed by stress-relaxation, sinusoidal, and ultimate tests (Figure 1). A zero-load length was established by applying tensile stress of 10 kPa. The sample was preconditioned with a 2% strain amplitude for 10 cycles using a velocity of (0.33% of sample length)/s, followed by a recovery before the execution of the elastic measurement protocol. The protocol consisted of a four-step stress-relaxation test with each step consisting of a 2% strain amplitude with a velocity of (20% of the sample length)/s, followed by 5 min relaxation time. After the fourth relaxation time, dynamic (sinusoidal) measurements were performed with 1 and 2% strain amplitudes with frequencies of 0.5, 1.0, and 2.0 Hz, followed by a sample relaxation at the zero-load length (15 min). The stress-relaxation protocol was used to determine equilibrium modulus as the ratio of stress and strain from relaxation timepoints, whereas dynamic modulus (ratio of stress and strain at same phase) along with storage and loss moduli, a phase difference (between stress and strain curves), and dissipated energy (area under fitted stress-strain curve) were determined from sinusoidal loading. Finally, the ultimate tensile test was carried out with a velocity of (0.33% of sample length)/s until breakage. In the ultimate test, several properties were determined for toe, linear, and failure regions. For the toe region, two fitting parameters, i.e., D and F, were determined according to Fung (1967) (Fung, 1967). When correlating WSSs and biomechanical measurements, planes one and two were used.

### 2.5 Statistical Analyses

Analyses were performed with MATLAB (The MathWorks, Natick, MA) or GraphPad Prism nine software (Dotmatics, Bishops Stortford, United Kingdom). Lilliefors test was used to determine the data distribution. When comparing the inner and outer curves, paired *t*-test was used with normally distributed values. Otherwise, a two-sided Wilcoxon signed-rank test was used. To compare TAV, BAV, and control groups, an unpaired *t*-test or Mann-Whitney *U*-test was used. Outliers were defined for normal distributions with Grubbs’ test and excluded from statistical analyses. Pearson (*r*) or Spearman (*ρ*) correlation tests were used based on data distribution. *p* < 0.05 was used to define the statistical significance.

### 2.6 Study Approval

The study was approved by the Research Ethics Committee of the Northern Savo Hospital District (permission number 200/2017) and had an organization permit from KUH for processing personal data. The study followed the declaration of Helsinki. A written informed consent was obtained from every patient.

## 3 Results

**FIGURE 2:**
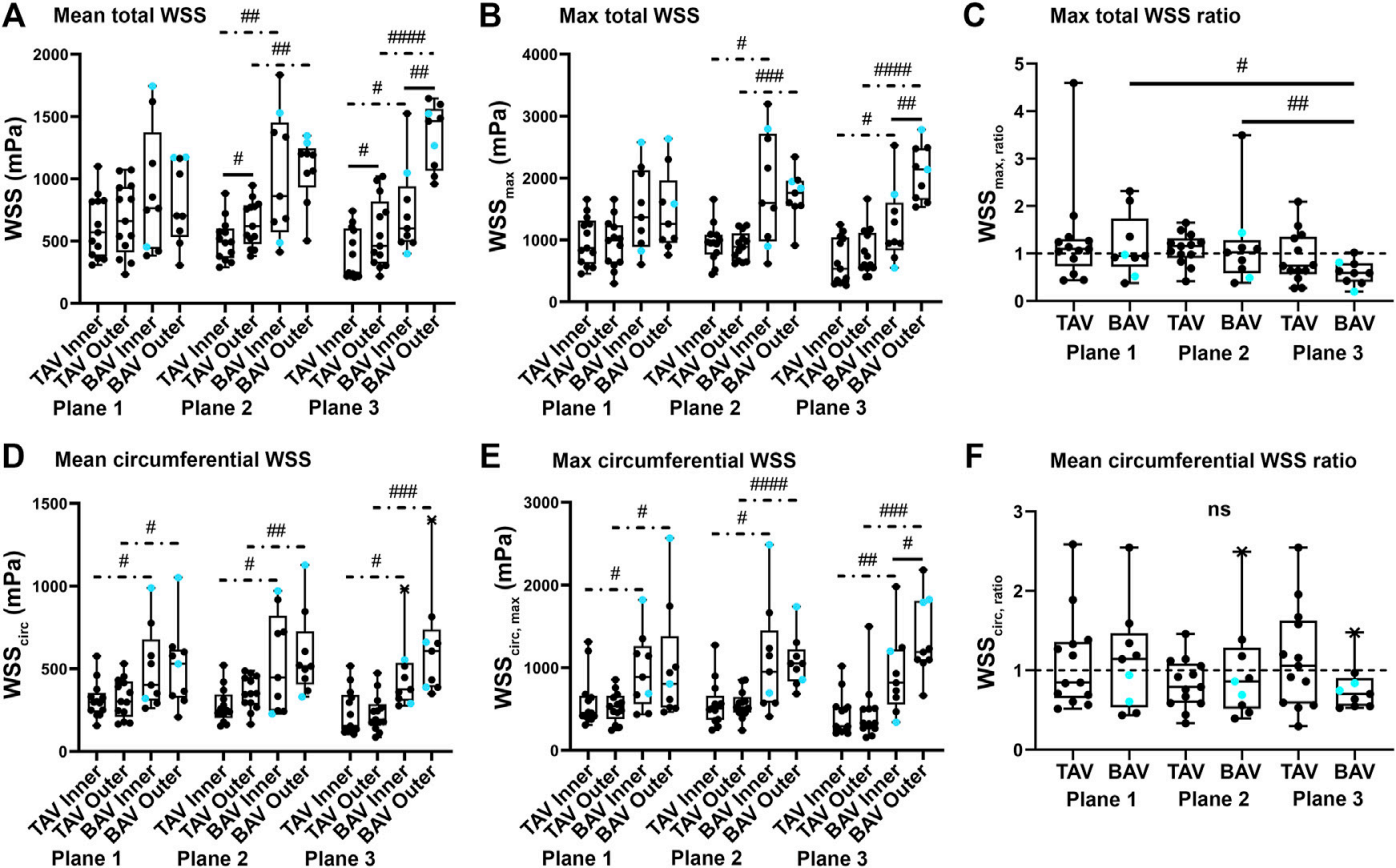
Total and circumferential (circ) wall shear stress (WSS, calculated with patient-specific viscosity) were evaluated on the three planes (with plane one closest to the aortic valve) separately for TAV and BAV patients. Boxplots of mean, maximum, and maximum ratio for total WSS [**(A–C), upper panels**] and similarly mean, maximum, and ratio for circumferential [**(D–F), lower panels**] WSSs were calculated and presented as median and outliers (a cross mark). BAV patients with aortic stenosis are presented with a turquoise circle. *p*-values < ^#^0.05, < ^##^0.01, < ^###^0.001, < ^####^0.0001. Hash sign: non-parametric test, solid line: paired experiment, dot-dash line: unpaired experiment.

WSSs (Figures 2A,B,D,E) of BAV patients were systematically greater compared to TAV patients regardless of the plane, curve, or statistic (mean and maximum). In addition, WSSs were greater in the outer curve. The patient-specific WSS correction had only minor effects on *p-*values when compared to those of non-corrected WSSs.

**FIGURE 3:**
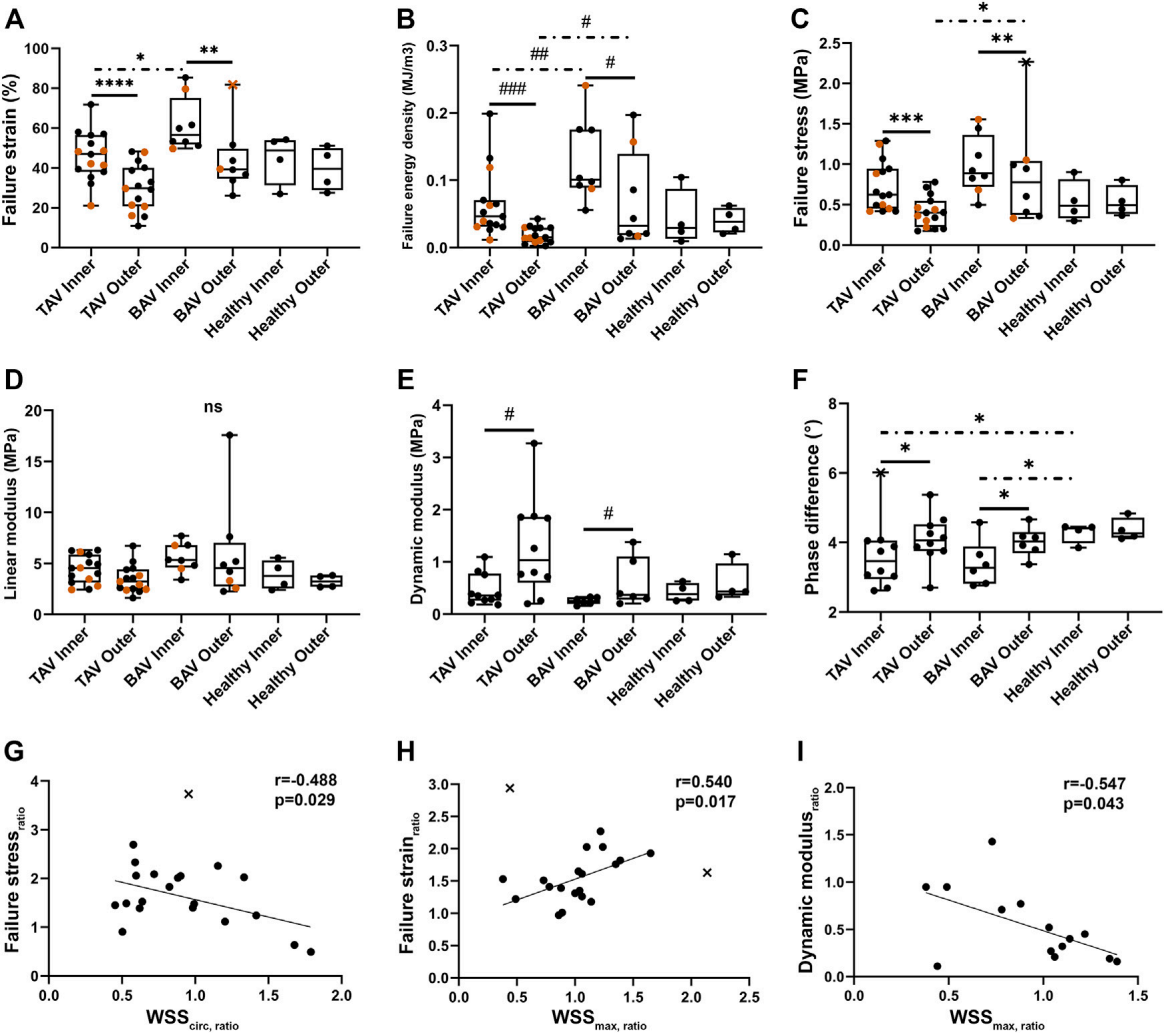
**(A–F)** Boxplots of the biomechanical properties for dilated AA with TAV or BAV, and healthy subjects with median and outliers (a cross mark). The black and vermilion circles present the 17N and 250N (less accurate) load cells, respectively. **(G–I)** Correlations were detected between circumferential WSS_ratio_ and failure stress **(G)** ratio and between maximum total WSS_ratio_ and failure strain **(H)** and dynamic modulus **(I)** ratios. *p*-values < *0.05, < **0.01, < ***0.001, < ****0.0001, ns not significant. Asterisk: parametric test, hash sign: non-parametric test, solid line: paired experiment, dot-dash line: unpaired experiment.

In biomechanical measurements, the most significant differences between inner and outer curves of the AA were observed in the ultimate test (Figures 3A–D, Supplementary Figures S1A–C) compared to the relaxation (Supplementary Figure S1D) and sinusoidal (2% strain and 1.0 Hz, Figures 3E,F, Supplementary Figures 1E–I) tests. The inner curves tolerated significantly higher failure strains (i.e., extension) and failure energy densities than the outer curves. Furthermore, TAV patients had significantly lower failure values than BAV patients (Figures 3A–C). Higher dynamic modulus and phase difference were observed with outer curves compared to inner curves (Figures 3E,F, Supplementary Figure S1D), denoting outer curves to be less elastic. The strain amplitude did not affect the phase difference, but with lower loading frequencies the tissue behaved more elastically (Supplementary Figure S1E). With controls, no differences were observed (Figure 3, Supplementary Figure S1). When correlating WSSs and biomechanical properties, ratios (i.e., inner curve value divided by outer curve value) were used with both parameters to limit the effect of patient-to-patient variability. The correlations between WSS and failure stress, failure strain, and dynamic modulus depict that the higher total and lower circumferential WSS_ratio_ predict that TAA is more susceptible to rupture (Figures 3G–I). For failure strain (Figure 3H), the similar trend was observed with both BAV and TAV groups (BAV: *r* = 0.460, *p* = 0.252, n = 8; TAV: *r* = 0.601, *p* = 0.051, n = 11), whereas with failure stress (Figure 3G, BAV: *r* = -0.231, *p* = 0.583, n = 8; TAV: *r* = -0.742, *p* = 0.006, n = 12) and dynamic modulus (Figure 3I, BAV: *r* = -0.958, *p* = 0.003, n = 6 TAV: *r* = -0.266, *p* = 0.524, n = 8) the trends were better held by TAV and BAV groups, respectively. Patient age correlated significantly with failure strain (*ρ* = -0.503, *p* = 0.0007, n = 42), dynamic modulus (*ρ* = 0.634, *p* = 0.0003, n = 28) and phase difference (*r* = 0.640, *p* = 0.0002, n = 28), depicting increasing age to decrease the strain required for rupture and the tissue to become less elastic. For BAV and TAV groups, the age-dependent trends of failure strain and dynamic modulus were similar, whereas with phase difference a stronger age-dependency was observed in TAV patients (BAV: *ρ* = 0.258, *p* = 0.418, n = 12; TAV: *r* = 0.804, *p* = 0.0002, n = 16).

**FIGURE 4:**
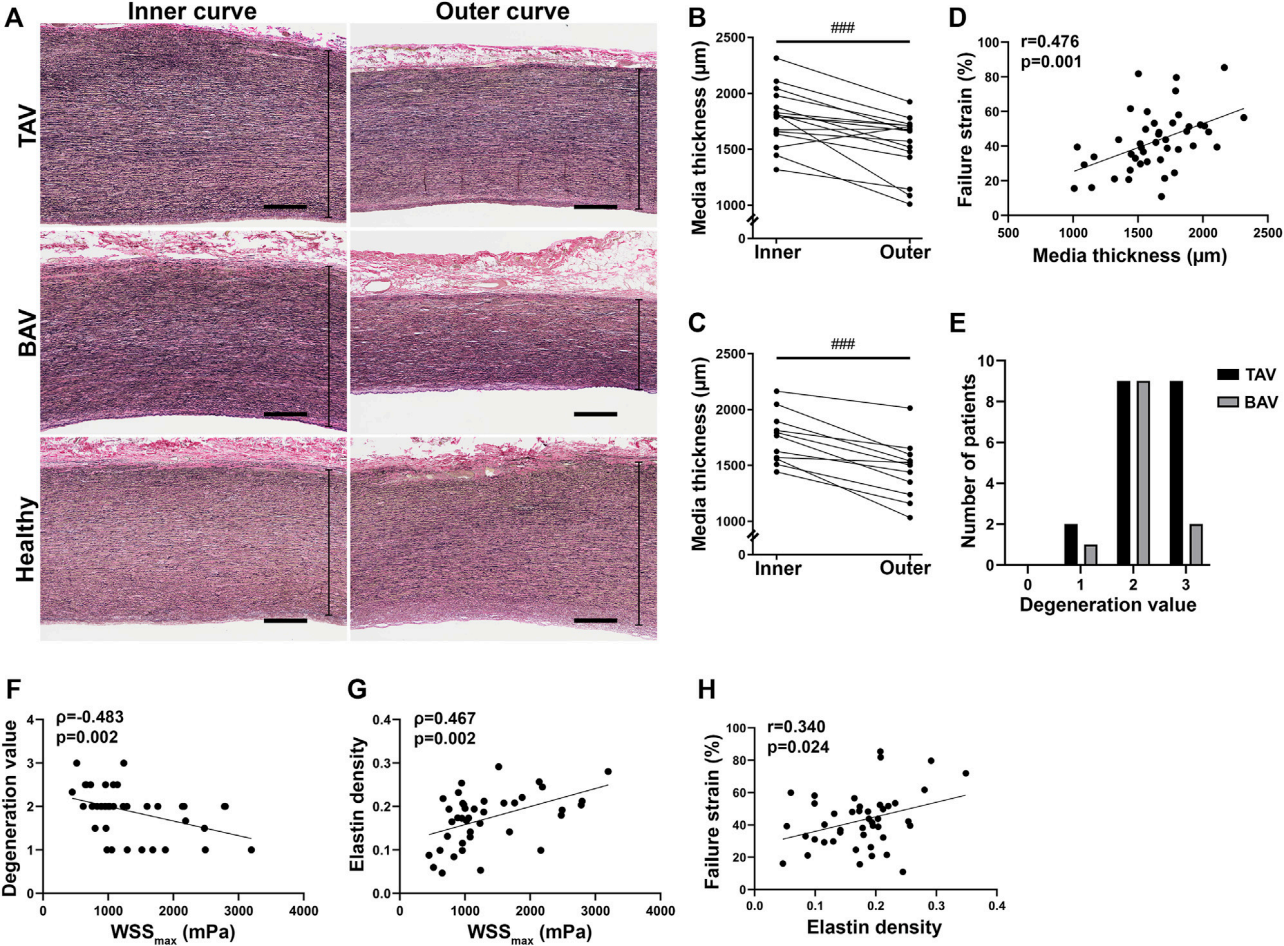
**(A)** Representative images of medial layer in the inner and outer curves. Scale bars, 500 µm. **(B,C)** Media thickness of the inner and outer curves of dilated AA with TAV **(B)** and BAV **(C)**. **(D)** Correlation was detected between media thickness and failure strain in all patients with TAA. **(E)** Moderate to severe media degeneration was observed in TAA patients. **(F,G)** Correlations were detected between maximum total WSS and media degeneration **(F)** and elastin density **(G)**. **(H)** Correlation was detected between elastin density and failure strain in all patients with TAA. *p*-values < ^###^0.001. Non-parametric test.

The media was significantly thinner in the outer curves than in the inner curves in patients with TAA (Figures 4A–C) and correlated with failure strain (Figure 4D). No difference was detected in controls. Moderate to severe media degeneration was observed in most TAA patients (Figure 4E, Supplementary Figures S2A,B). However, no difference was detected between the inner and outer curves. Instead in TAV patients, elastin density was significantly decreased in the outer curves (*p* = 0.012) (Supplementary Figure S2C). Correlations between WSS and media degeneration or elastin density depicted that lower WSS predicts more severe pathological changes of the AA (Figures 4F,G). As expected, reduced elastin level led to a weakened aortic wall and lower failure strain (Figure 4H). Based on H&E and elastin stainings, the structure of the aortic media was normal in controls. No significant age-dependent correlation was found with histology.

**FIGURE 5:**
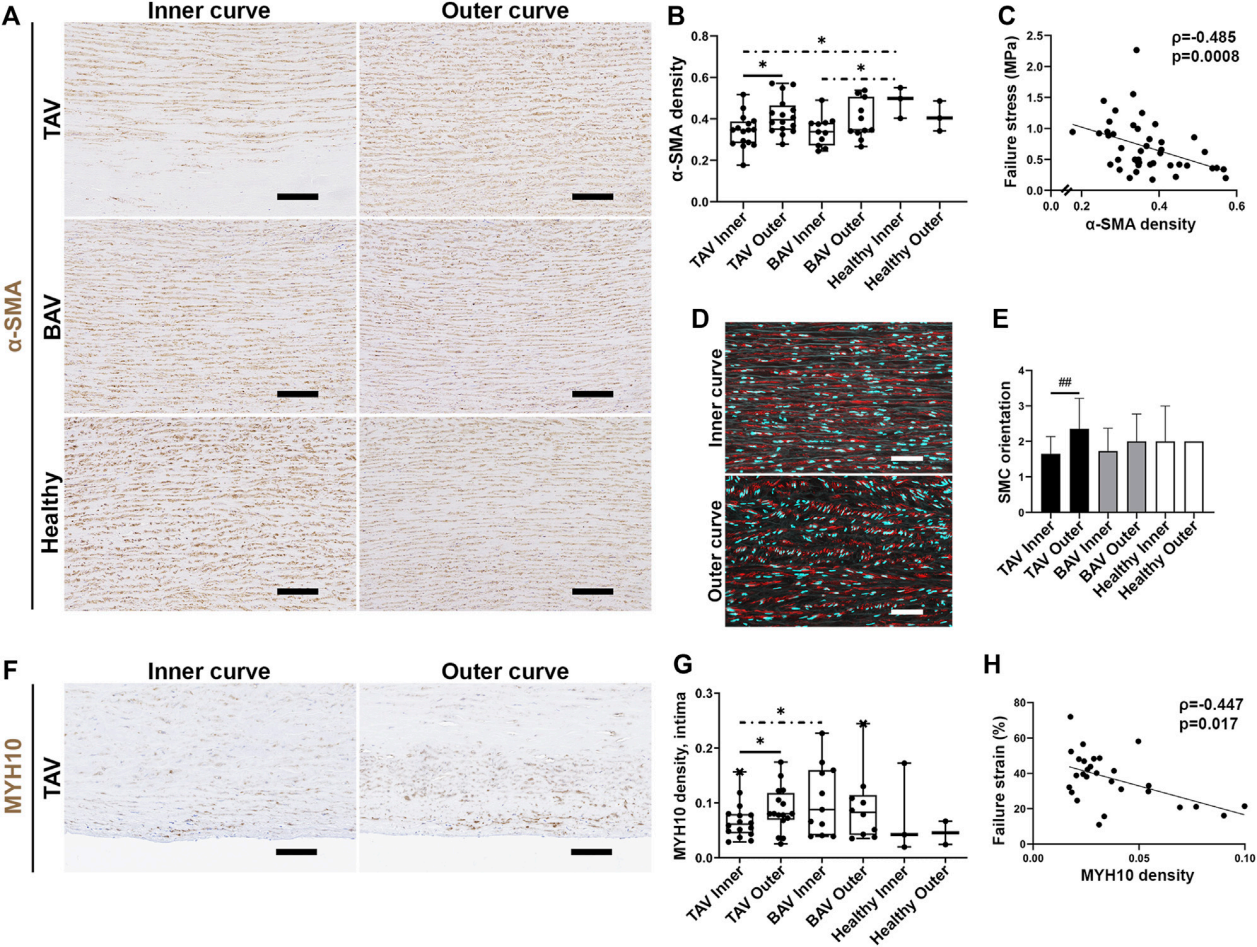
**(A)** Representative images of contractile SMCs in the inner and outer curves. Scale bars, 200 µm. **(B)** The density of α-SMA^+^ cells was increased in the outer curves in TAV patients. α-SMA density was higher in the inner curves of healthy aortas compared to patients with TAA. **(C)** Correlation was detected between α-SMA density and failure stress in all patients with TAA. **(D)** Representative images of SMC orientation in the inner and outer curves in TAV patients (red, α-SMA; cyan, DAPI-labeled nuclei). Scale bars, 100 µm. **(E)** SMC orientation was changed from linear to random in the outer curves in TAV patients. **(F)** Representative images of MYH10 staining in TAV patients. Scale bars, 100 µm. **(G)** MYH10 density was increased in the intimal layer of the outer curves in TAV patients. **(H)** Correlation was detected between MYH10 density and failure strain in TAV patients. *p*-values < *0.05, < **0.01. Asterisk: parametric test, hash sign: non-parametric test, solid line: paired experiment, dot-dash line: unpaired experiment.

Increased density (Figures 5A,B) and random orientation (Figures 5D,E) of the contractile α-SMA^+^ SMCs were detected in the outer curves of TAV patients, whereas in the inner curves, cells were mostly organized in linear orientation (Figures 5D,E). A correlation was detected between α-SMA density and failure stress (Figure 5C). No change was observed in the number of contractile SMCs, between the inner and outer curves (Figure 5A, Supplementary Figure S3A), or in the number of SMC nuclei in media (Supplementary Figure S3B). MYH10 expressing cells, representing a synthetic proliferative SMC phenotype (Li et al., 2020), were observed in the media in patients with TAA (Supplementary Figures S3C–E). Particularly, in the outer curves of TAV patients MYH10 density was increased (*p* = 0.047) (Figures 5F,G), and correlated with failure strain (Figure 5H), indicating the more MYH10 expression the lower the strength of the aortic wall. No statistically significant changes in SMC density, orientation or MYH10 expression were seen in BAV patients.

**FIGURE 6:**
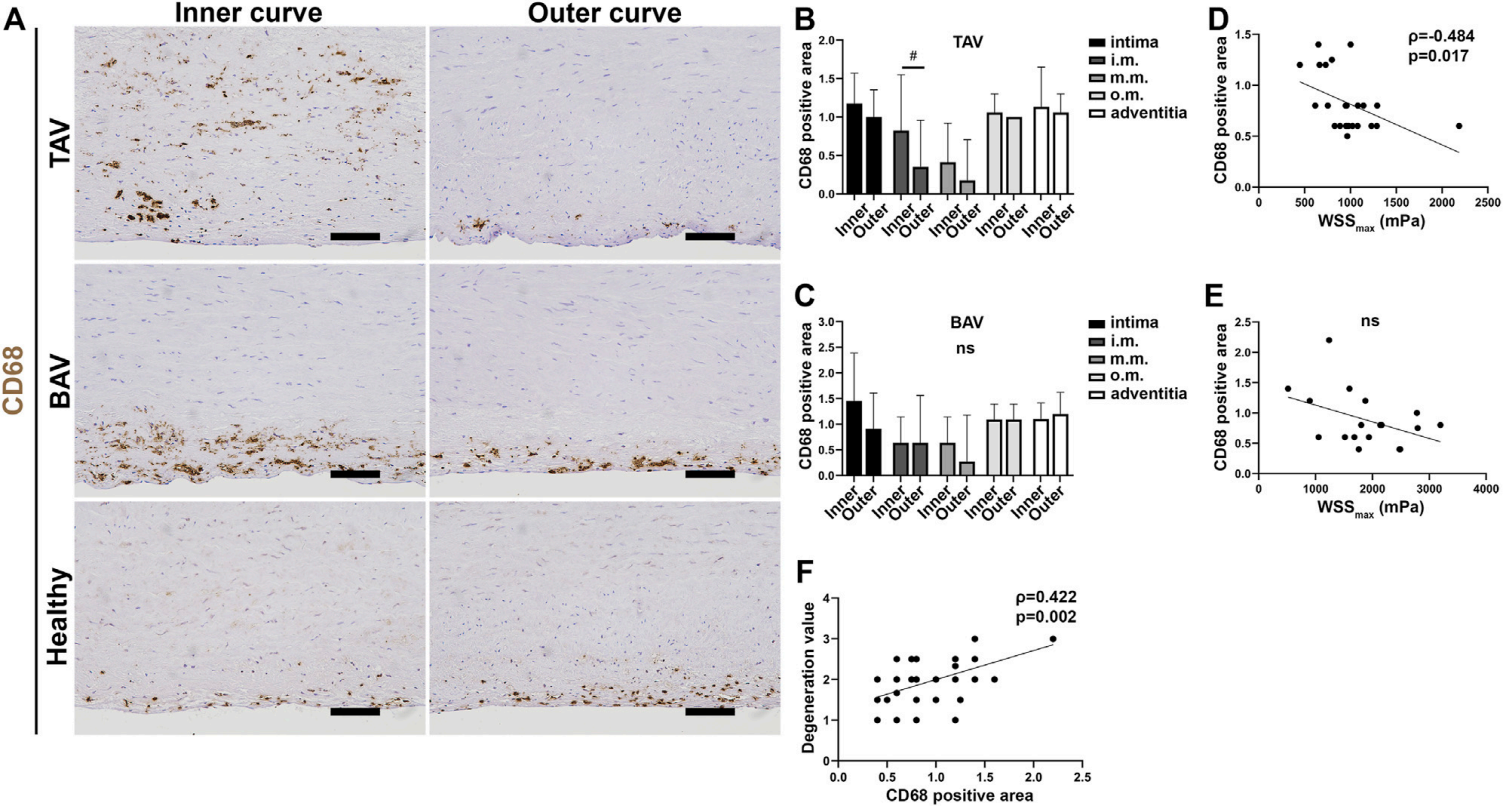
**(A)** Representative images of CD68^+^ macrophages in patients with TAA. Scale bars, 100 µm. **(B,C)** CD68^+^ area scoring in TAV **(B)** and BAV **(C)** patients. In TAV patients, increase of macrophages was detected in the inner curves of the AA in the innermost part of the medial layer. **(D,E)** Correlation was detected between CD68^+^ area and maximum total WSS in TAV **(D)** but not with BAV **(E)**. **(F)** Correlation was detected with media degeneration score and CD68^+^ area in all patients with TAA. *p*-values < ^#^0.05, ns not significant. Non-parametric test.

Inflammatory cells located mostly in the intima, outer media, and adventitia in patients with TAA detected by H&E staining (data not shown). No difference was detected in the overall inflammation of the aortic wall between TAV and BAV patients (data not shown). In patients with TAA, CD68^+^ areas were detected in the intima, outer media, and adventitia. In TAV, the area of CD68^+^ cells was increased in the inner media of the inner curves (*p* = 0.035) (Figures 6A,B). The same tendency was detected in BAV patients (*p* = 0.125; Figure 6C). Correlation between WSS and CD68^+^ cells indicated that with lower WSSs more CD68^+^ cells, determined by their shape and size as macrophages, infiltrated to the aortic wall (Figures 6D,E). The amount of CD68^+^ cells also correlated with the media degeneration (Figure 6F), indicating the more inflammatory cells the more severe media degeneration. No differences were detected in CD3^+^ T cells between the inner and outer curves or between TAV and BAV patients (data not shown).

## 4 Discussion

Predicting and preventing aortic dissections and ruptures continues to be challenging, and more accurate assessment of the AA dilatation, identification of high-risk patients, and timing of repair surgery are required. This is the first study investigating the crucial aspects of aortic wall remodeling in TAA including biomechanics, WSS, media degeneration, and cell content in the same patient cohort. We demonstrate that MRI-derived absolute WSS and WSS_ratio_ are predictive of tissue remodeling, thereby enabling the identification of high-risk patients. Our study also provides valuable knowledge of aneurysm pathobiology in TAA.

Previously, vascular flow has been suggested to induce changes in the aortic wall and to cause aneurysm formation or progression. Regional increases in WSS and disturbed flow have been detected in TAA (Mahadevia et al., 2014; van Ooij et al., 2017; Rodríguez-Palomares et al., 2018; Kauhanen et al., 2019). As examples, higher circumferential WSS values of BAV patients (Bollache et al., 2018; Rodríguez-Palomares et al., 2018), and lower WSS values in TAV patients (Bürk et al., 2012; van Ooij et al., 2015; Kauhanen et al., 2019). Similarly, to this study, higher total WSS values for BAV patients compared to TAV patients have been reported (Barker et al., 2012; Meierhofer et al., 2013; Mahadevia et al., 2014; van Ooij et al., 2017).

The relationship between WSS and tissue’s biomechanical properties is not well known. Generally, aortic wall degeneration is considered to lead to decreased ultimate strength (Vorp et al., 2003). Salmasi et al. and Condemi et al. did previously present that high time-averaged WSS (TAWSS) values are predictive of the higher ultimate tensile strength (coefficient = 0.136, *p* = 0.048, n = 63, and r = 0.818, *p* = 0.004, n = 10, respectively; Salmasi et al., 2021; Condemi et al., 2020)*.* Interestingly, Condemi et al. (Condemi et al., 2020) instead did not find statistical significance with WSS derived at peak systole as in this study. Similarly, to previous studies (Ferrara et al., 2016; Salmasi et al., 2021), we found that the inner curves of the AA had higher failure values (i.e., strength and extension) compared to the outer curves, and that BAV patients tend to have significantly higher failure statistics than TAV patients (García-Herrera et al., 2012; Pham et al., 2013; Pichamuthu et al., 2013). The wall thickness was also shown to correlate with the failure values by Iliopoulus et al. (Iliopoulos et al., 2009a) alike to our study, and similar age-related differences in biomechanical properties have previously been presented by Okamoto et al. (2002); Duprey et al. (2016). However, in contrast to these, no previous study utilized relaxation and sinusoidal tests in their analysis, arguably due to the lengthy protocol. Overall, the ultimate tensile test was found here to be the most descriptive of the biomechanical properties of the AA with the sinusoidal loading providing additional information. Most intriguingly, the WSS_ratio_ was determined as a valuable predictor for estimating tissue’s susceptibility to rupture. Specifically, the parameter describes the relative differences experienced by the aortic wall and limits the role of absolute WSS magnitude, thereby better describing flow eccentricity and disturbances.

Reduced levels of elastin and increased aortic stiffness have previously been observed in TAA (Iliopoulos et al., 2009b). In our study, a positive correlation was observed between elastin and failure strain. Decreased elastin content and increased aortic rigidity were particularly observed in the outer curves of the dilated AA. Additionally, correlations between WSS and elastin level or media degeneration, were observed indicating that lower WSSs predict greater pathological changes of the AA. Prior to our study, WSSs have not been associated with degenerative aortic histopathology score, a standardized criteria to determine pathological changes in the AA (Halushka et al., 2016). On the contrary to our data, Salmasi et al. (2021) and Guzzardi et al. (2015) demonstrated earlier a correlation between high maximum WSS and low elastin content in TAA in BAV or TAV patients. However, compared to our study, elastin content was measured in these studies either from different segments of the AA or from a smaller area, the samples were frozen prior to fixation or a different image analysis method was used to determine the correlation. Also, Bollache et al. (2018) demonstrated a low correlation (*r* = -0.25, *p* = 0.02) with elastic fiber thinning and increased WSS in TAA patients with BAV. However, the study did not use a comprehensive, standardized histopathology scoring as we did from 10–25 areas per patient (depending on the size of the AA) and did not analyze TAA patients with TAV.

Besides extracellular matrix (ECM) components, SMCs have been suggested to regulate aortic stiffness (Sehgel et al., 2015). Recently, multiple SMC phenotypes and immunomodulatory cells were observed in TAA by single-cell RNA-sequencing (Li et al., 2020; Jauhiainen et al., 2022). However, the role or location of these cell phenotypes in TAA have remained unknown in relation to WSS or aortic strength (Jauhiainen et al., 2022). In our study, a changed SMC orientation and increased expression of a SMC marker MYH10 were detected in TAV patients. Our data indicated the more MYH10, the lower the strength of the aortic wall, thus suggesting that MYH10 could be a potential marker for detection of changes in the aortic wall in TAA patients. As the change in ECM proteins has been associated with SMC phenotype switching (Qin et al., 2000), the presence of synthetic/proliferative SMCs could be associated with the increased rigidity of the aortic wall. Interestingly, no differences in SMC orientation or MYH10 expression were observed in BAV patients. Although studies have indicated clear differences in the aortic wall structure, TGF-β signaling, and SMC composition and signaling between TAV (proliferative SMCs) and BAV (senescent SMCs) patients with TAA (Balistreri et al., 2013; Paloschi et al., 2015; Ignatieva et al., 2017), genetic and cellular differences between these entities are still largely uncharacterized.

Previously, macrophages and SMCs have been suggested to express elastolytic proteinases, such as matrix metalloproteinases (MMP) (Newby, 2006) that could affect aortic rigidity. MMP secreting macrophages have been associated with abdominal aortic aneurysm (Thompson et al., 1995). In our study, infiltrated macrophages located predominantly in the inner curves of the AA which had lower WSSs. Although increased amounts of immune cells have previously been found in the medial layer of the dilated AA in comparison to healthy AA (He et al., 2008), their presence has not been associated with lower WSS prior to our study. As we observed increased aortic rigidity particularly in the outer curves of the dilated AA, the relation between site-specific macrophages and changes in the aortic wall in the inner curves requires further studies.

The main limitation of this study is the limited number of healthy subjects hindering the statistical power of the comparison of the histological data; however, as obtaining fresh aortic samples from organ donors with no cardiovascular diseases is rare, these samples are nevertheless highly valuable. Also, as TAV patients were significantly older than BAV patients at the time of surgery, this can affect our results. A significantly faster growth rate of the AA dilatation in BAV patients at a younger age has been previously demonstrated (Tadros et al., 2009).

Currently, surgical evaluation is moving towards more personalized risk assessment based on patient size and indicators of cardiovascular health, such as blood pressure (Hiratzka et al., 2010; Erbel et al., 2014). No previous study has investigated the relationship between WSS, tissue’s biomechanical properties, media degeneration, and cellular remodeling in the same patient cohort. Our data revealed for the first time the relation of WSS, failure strain, and site-specific cell composition in TAA emphasizing the role of WSS and WSS_ratio_ in regulating both biomechanical properties, aortic degeneration, and cell-driven mechanisms. We also identify MYH10 as a potential marker to indicate changes in aortic wall structure in TAA in patients with TAV. From a clinical perspective, our data gives insights why the outer curve of the AA is more prone to rupture in TAA as demonstrated in earlier studies (Murray and Edwards, 1973; Akashi et al., 2003). This might indicate that MRI-derived parameters, such as WSSs, could be used as predictors for pathological changes of the aortic wall, enabling better identification of high-risk TAA patients.

## Data Availability

The original contributions presented in the study are included in the article/Supplementary Material, further inquiries can be directed to the corresponding author.
