# Supplementary figures and images for: Wall Shear Stress Predicts Media Degeneration and Biomechanical Changes in Thoracic Aorta
Source: Front Physiol. 2022 Jul 7;13:934941. doi: 10.3389/fphys.2022.934941 (PMC9301078; doi:10.3389/fphys.2022.934941)

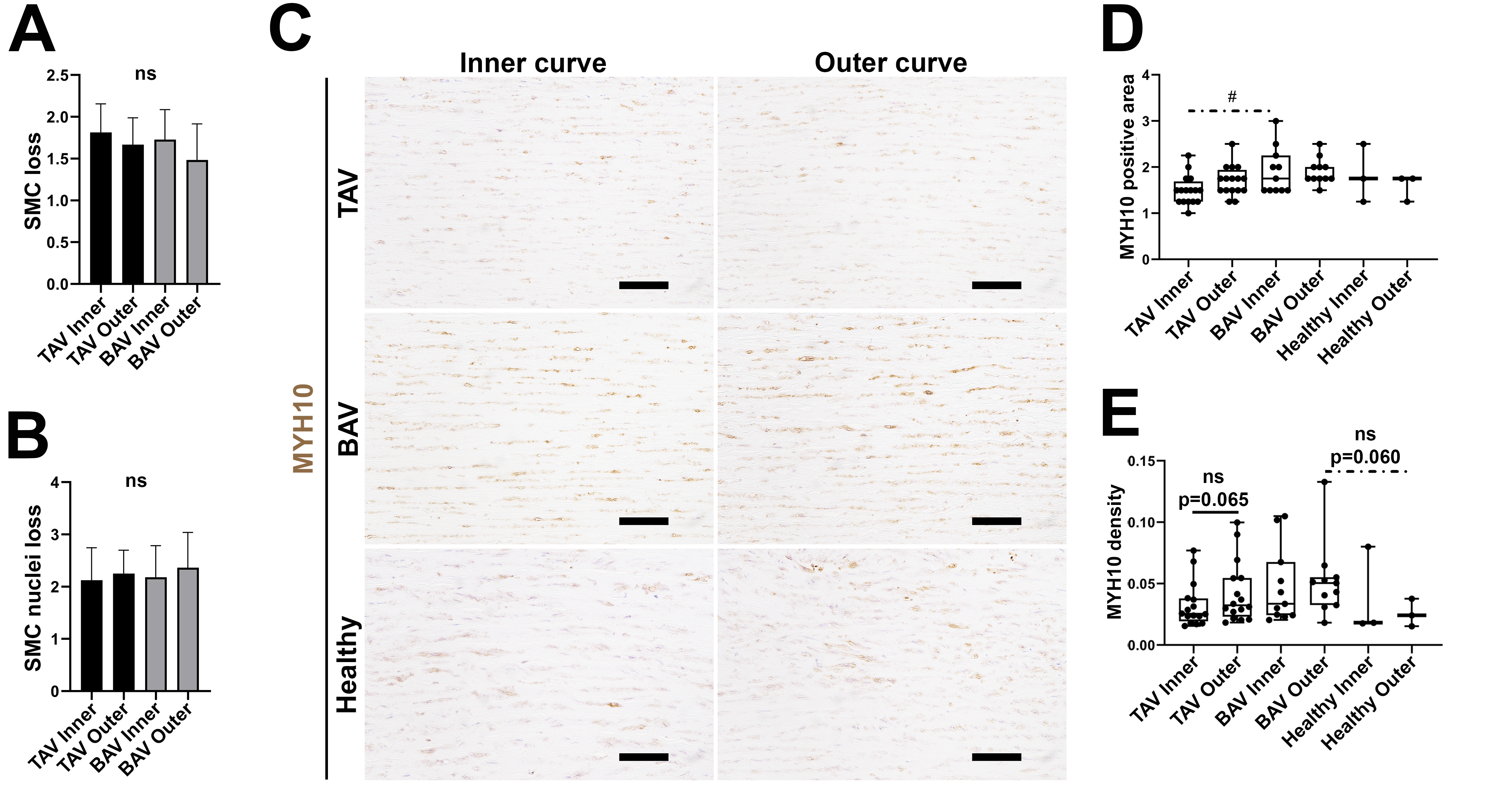

Supplement: Supplementary file 1 [file Image3.TIF]

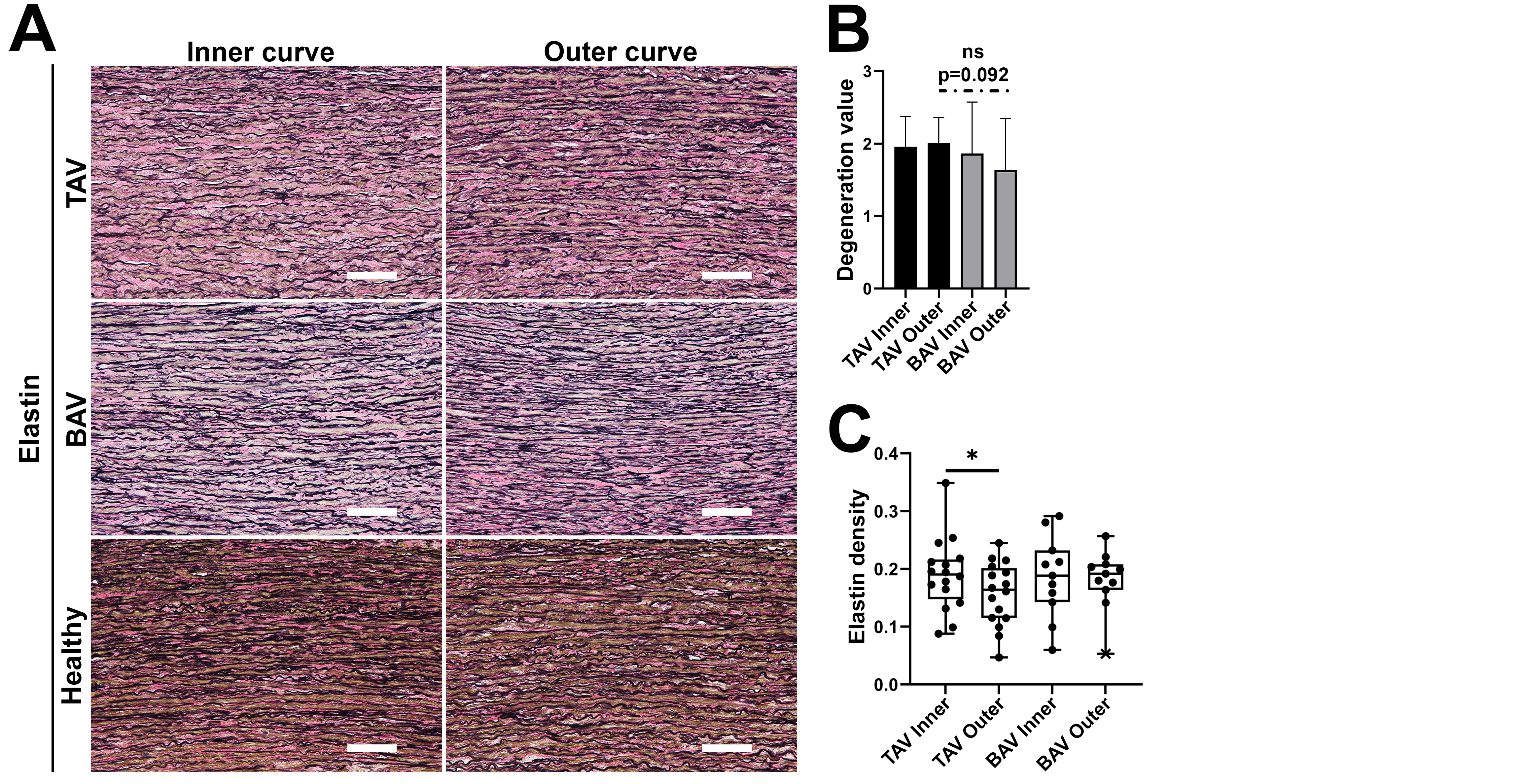

Supplement: Supplementary file 2 [file Image2.TIF]

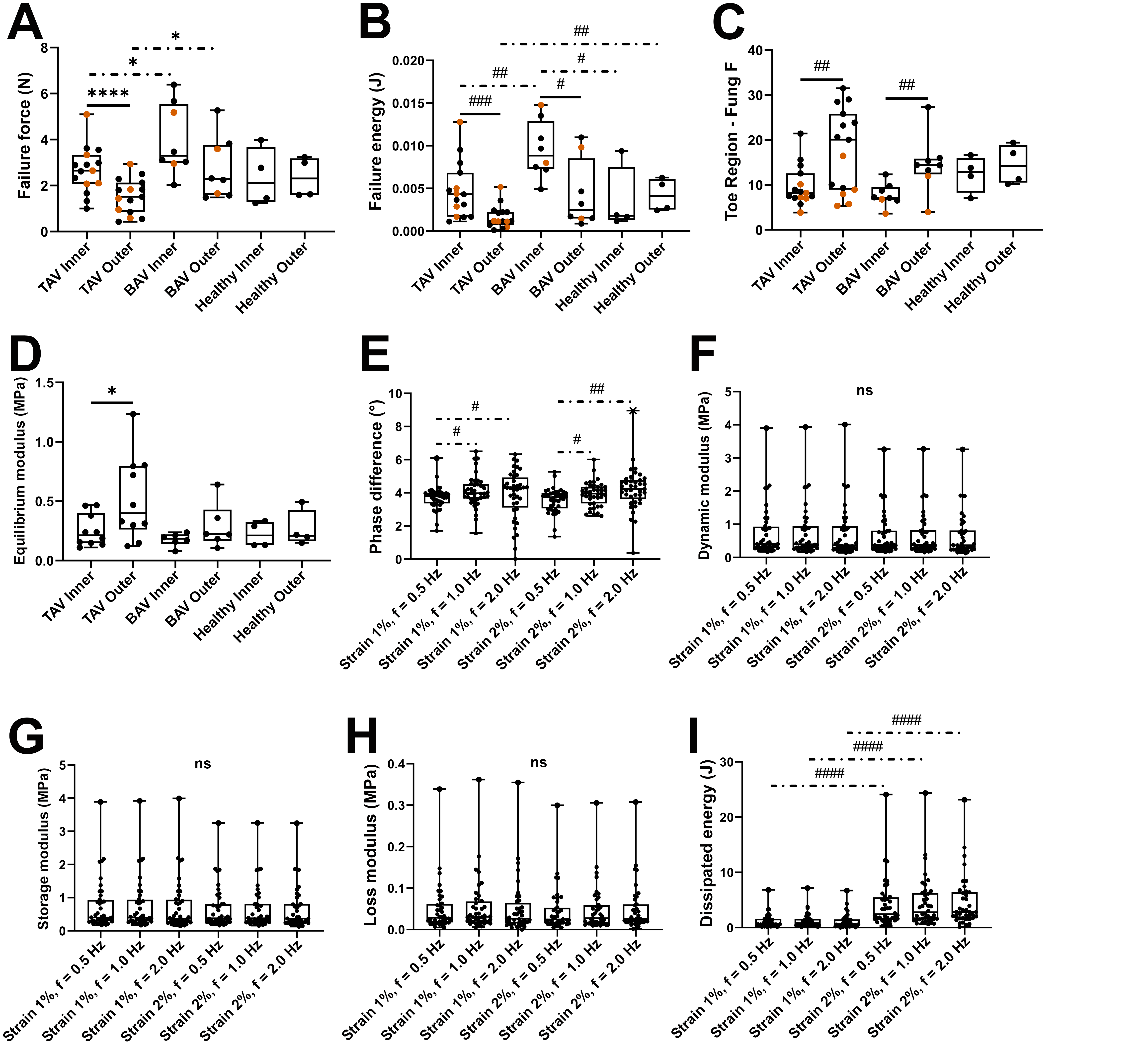

Supplement: Supplementary file 3 [file Image1.TIF]
