# Supplementary material for: Wall Shear Stress Predicts Media Degeneration and Biomechanical Changes in Thoracic Aorta
Source: Front Physiol. 2022 Jul 7;13:934941. doi: 10.3389/fphys.2022.934941 (PMC9301078; doi:10.3389/fphys.2022.934941)
Supplement: Supplementary file 4 [file DataSheet1.docx]

**SUPPLEMENTAL FIGURE LEGENDS**

**Suppl. Figure 1. A-D)** Boxplots of the biomechanical properties from the ultimate (**A-C**) and the relaxation (**D**) tests for dilated AA with TAV or BAV, and healthy subjects with median. The black and vermilion circles present the 17N and 250N (less accurate) load cells, respectively. **E-I)** Boxplots of the sinusoidal loading properties for different strains and frequencies with outliers (a cross mark). *P*-values < *0.05, < **0.01, < ***0.001, < ****0.0001, ns not significant. Asterisk: parametric test, hash sign: non-parametric test, solid line: paired experiment, dot-dash line: unpaired experiment.

**Suppl. Figure 2. A)** Representative images of elastin staining (black) in the inner and outer curves of the AA in patients with TAA. Scale bars, 100 µm. **B)** No difference in media degeneration was observed between the inner and outer curves of dilated AA in patients with TAA. **C)** Elastin density was decreased in the outer curves in TAV patients. *P*-values < *0.05, ns not significant. Parametric test.

**Suppl. Figure 3. A-B)** No differences were observed in contractile SMC loss detected by α-SMA (**A**) or SMC nuclei loss (**B**) between the inner and outer curves in patients with TAA. **C)** Representative images of MYH10 staining in the inner and outer curves. Scale bars, 100 µm. **D)** The number of MYH10^+^ cells was increased in the inner curves of BAV patients compared to TAV patients. **E)** No difference in MYH10 density was observed between the inner and outer curves of dilated AA in patients with TAA. *P*-values < ^#^0.05, ns not significant. Non-parametric test.
